# Supplementary material for: Persistence of Untreated Bed Nets in the Retail Market in Tanzania: A Cross-Sectional Survey
Source: Trop Med Infect Dis. 2025 Jun 19;10(6):175. doi: 10.3390/tropicalmed10060175 (PMC12197522; doi:10.3390/tropicalmed10060175)
Supplement: Supplementary file 1 [file tropicalmed-10-00175-s001.zip › tropicalmed-3645240-supplementary.pdf]

Supplementary material: Sensitivity analysis of the weighting approach used for market share

Figure S1, Panel a: Market share weighted by mid-point value of sales category

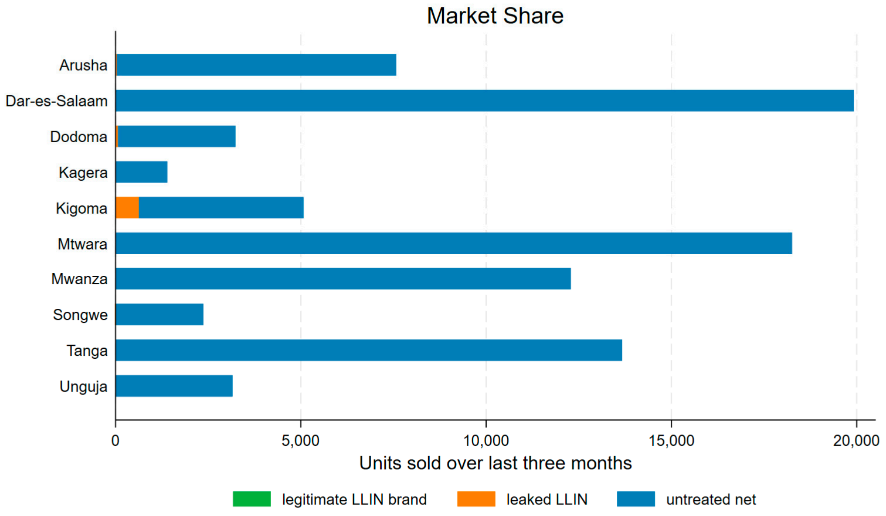

Figure S1, Panel b: Market share weighted by integer sales

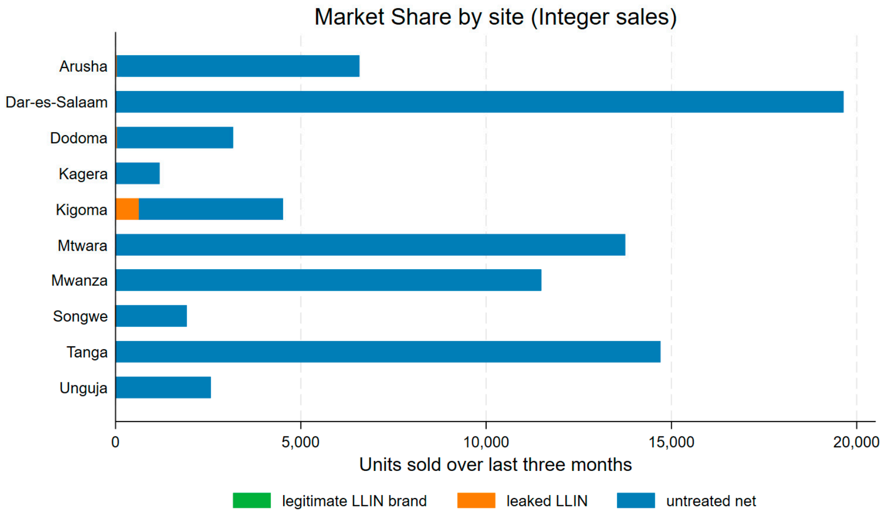

Differences in market share values are observed for untreated nets in Mtwara region, where 18,255 sales are estimated using the mid-point category weight compared to 13,755 sales when using the integer sales data. These differences are largely driven by 4 cases with integer sales of 550, 1000, 1000, and 1000 units, which are inflated when using the mid-point category value. Across all regions, 86,195 sales of untreated nets are estimated using the mid-point category weight compared to 78,846 sales using the integer data; this represents a possible overestimate of 9% when compared to the integer data.
